# Protein expression guided chemical profiling of living cells by the simultaneous observation of Raman scattering and anti-Stokes fluorescence emission

**DOI:** 10.1038/srep43569

**Published:** 2017-03-08

**Authors:** Liang-da Chiu, Taro Ichimura, Takumasa Sekiya, Hiroaki Machiyama, Tomonobu Watanabe, Hideaki Fujita, Takeaki Ozawa, Katsumasa Fujita

**Affiliations:** 1 Department of Chemistry, the University of Tokyo, Tokyo, Japan; 2 Department of Applied Physics, Osaka University, Osaka, Japan; 3 Quantitative Biology Center, RIKEN, Osaka, Japan; 4 Immunology Frontier Research Center, Osaka University, Osaka, Japan

## Abstract

Our current understanding of molecular biology provides a clear picture of how the genome, transcriptome and proteome regulate each other, but how the chemical environment of the cell plays a role in cellular regulation remains much to be studied. Here we show an imaging method using hybrid fluorescence-Raman microscopy that measures the chemical micro-environment associated with protein expression patterns in a living cell. Simultaneous detection of fluorescence and Raman signals, realised by spectrally separating the two modes through the single photon anti-Stokes fluorescence emission of fluorescent proteins, enables the accurate correlation of the chemical fingerprint of a specimen to its physiological state. Subsequent experiments revealed the slight chemical differences that enabled the chemical profiling of mouse embryonic stem cells with and without *Oct4* expression. Furthermore, using the fluorescent probe as localisation guide, we successfully analysed the detailed chemical content of cell nucleus and Golgi body. The technique can be further applied to a wide range of biomedical studies for the better understanding of chemical events during biological processes.

Non-targeted biochemical analysis is gaining attention in recent years^1^,^2^. This is because it provides a more general picture about the overall metabolic flux ongoing inside a specimen by visualising its whole biochemical profile instead of focusing on specific bio-molecules. Liquid chromatography combined with mass spectrometry (LC/MS) has been the gold standard for such analysis due to its high molecular specificity and accuracy in quantification. However, the requirement of a large amount of cells and its destructive nature made LC/MS difficult to visualize the individual differences of cells and their time-dependent changes, which is necessary to study the gain or loss of cell functions. To better address the biochemical dynamics in living cells, Raman spectroscopy has also become an expected tool, because it is a non-destructive and label-free technique that can analyse the biochemical content of living cells at a sub-cellular resolution^3^,^4^,^5^. The wide application of Raman spectroscopy in actual cancer surgery of human patients also indicates that the method is biologically safe^6^,^7^. One major challenge for the Raman related biomedical studies is the biological validation of the profiled chemical pattern^8^. To address this challenge, here we introduce a new hybrid fluorescence-Raman microscopy method for the simultaneous chemical profiling by Raman spectroscopy and identification of cell state by fluorescence imaging. Fluorescence microscopy has long been a major technique for the study of cellular dynamics^9^,^10^. However, the simultaneous acquisition of the both the fluorescence and the Raman modes has proven to be a very challenging task, because fluorescence signals easily overlap with Raman signals so that the Raman signals are usually buried under the much stronger fluorescence signal.

So far, beside the use of fluorescence intensity as single-spot Raman measurement guide^11^, successful reports on the hybrid Raman-fluorescence imaging of cells are limited to the two-photon excitation of fluorescent probes with higher two-photon absorption cross-section, such as quantum dots^12^ or organic dyes^13^. This is because under normal Raman measurement condition, the quantum efficiency of FPs is not enough for two-photon excitation to occur. However, genetically-encoded FPs can hardly be replaced by the other probes in terms of its unparalleled specificity by tagging its target with a covalent bond^9^, and they have seen a much wider range of applications than the other fluorescent probes, ranging from the study of protein dynamics^14^,^15^,^16^, cell state inditation^17^, to sensing a specific chemical parameter in cells^18^,^19^. It is therefore of utmost importance to properly combine Raman and FP detection for the analysis of biological specimen. Unfortunately, due to the difficulty of integrating Raman and FP detection, previous reports either use the FP image as a guide for single spot Raman spectroscopy^20^, or they acquire the FP image and Raman image separately then use calculation methods for colocalisation analysis of the two dataset^21^. These methods either lack the full chemical information across the specimen, or fail to acquire the fluorescence and Raman information simultaneously, which is not suitable for the study of dynamic samples such as living cells. At the meanwhile, although the combination of coherent Raman microscopy with the two-photon fluorescence imaging of fluorescent proteins has seen significant progress^22^, due to the high hurdles in obtaining the spectral information with coherent Raman microscopy (either technical-wise or cost-wise), spontaneous Raman microscopy is still the standard tool for multivariate chemometrics analysis of living cells, Therefore, developing an easy-to-implement and cost-efficient method to combine fluorescent protein and Raman spectral imaging is the key to help elucidate the correlation between the protein expression pattern and the chemical profile in living cells.

Here, we exploit anti-Stokes fluorescence emission to realise the simultaneous imaging by fluorescence and Raman scattering. Anti-Stokes fluorescence emission is a single photon excitation process of a fluorophore at the long wavelength tail of its excitation spectrum, so that even the emission peak is at the short wavelength side of the excitation light (Fig. 1). This excitation method leaves an around 100 nm wavelength window at the Stokes side of the excitation light for the combination of various optical techniques, including Raman spectroscopy. The implementation is also extremely simple: all that is required is to replace the long pass filter in front of the spectrometer to a notch filter. The combination of genetically-encoded FP and Raman imaging would enable the correlation of a specimen’s Raman spectral pattern, or chemical profile, to its physiological state that were only accessible by fluorescent imaging techniques. Raman spectroscopy is known to be advantageous in the label-free and real-time chemical profiling of living systems, but the detailed chemical interpretation of the Raman spectra has always been difficult. Our method will directly provide the physiological meaning of the complicated Raman spectra taken from living cells, and circumvent this major bottleneck for the use of Raman spectroscopy in biomedical studies. In this report, our results demonstrated the successful chemical profiling of embryonic stem (ES) cells with and without Oct-4 expression.

In addition to the FP-guided chemical profiling of cells, once the chemical information of the Raman spectra can be deciphered, our method will further contribute to the analysis of tiny chemical differences between subcellular compartments that could hardly be discriminated solely by unsupervised multivariate spectral analysis of the Raman spectra without the FP guide. Here, we will also show the Raman spectral analysis of cell nucleus and Golgi body by our method. Recent advancements in biomedical studies had made clear that the metabolic environment is also an important factor in regulating cell behaviour^23^,^24^. Being capable of simultaneously detecting the metabolome profile and protein expression pattern, our method also has the potential to contribute to the non-targeted study of metabolic checkpoints for cell signalling cascades once multivariate spectral analysis methods are precise enough to extract enough chemical details from the observed specimen.

## Results

### Anti-Stokes fluorescence excitation of fluorescent proteins

**Figure 2:**
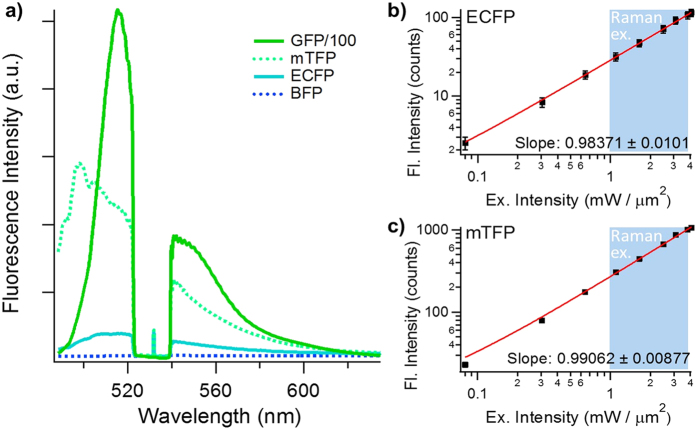
(**a**) The anti-Stokes fluorescence emission spectra of BFP, ECFP, mTFP and GFP. The spectral valley around 532 nm is due to the notch filter, and the weak remaining of Rayleigh scattering at 532 nm can still be seen at the centre of the spectral valley. Note that the excitation intensity for GFP is 100 times lower than the other FPs, because the emission spectrum for GFP would saturate otherwise. (**b**,**c**) shows the fluorescence response according to excitation intensity of ECFP and mTFP. Both of them shows a slope at around 1 in the log plot, which indicates the anti-Stokes excitation of FPs is a single-photon process. Standard deviation bars of each spot are from 3 repeated measurements. The blue region between 1~4 mW/μm^2^ excitation intensity is the common condition to measure the Raman spectra of cells.

Despite several scattered reports on the anti-Stokes fluorescence emission from chloroplasts^25^, organic^26^,^27^ and inorganic dyes^28^, to the best of our knowledge, the anti-Stokes fluorescence excitation of FPs has never been reported before. To verify whether the anti-Stokes emission from FPs can be detected under Raman measuring conditions, we recorded the emission spectra of various FPs with a shorter emission peak than the continuous-wave (cw) 532 nm laser that we use for our Raman experiments (Fig. 2a). For the FPs with emission spectra much shorter than 532 nm, i.e. blue fluorescent protein (BFP, emission peak at 445 nm), almost no fluorescence signals can be detected. However, for FPs with a closer emission spectra to 532 nm, i.e. enhanced cyan fluorescent protein (ECFP, emission peak at 476 nm), monomer teal fluorescent protein (mTFP, emission peak at 492 nm) and green fluorescent protein (GFP, emission peak at 509 nm), an obvious fluorescence emission peak can be seen at the anti-Stokes side of the spectra. One point that is worth mentioning is the absence of resonance enhanced Raman peaks of the fluorescent protein chromophore at the Stokes region of the spectra. This proves that our anti-Stokes fluorescence excitation method will introduce minimum perturbation to the Stokes spectral window that is left for Raman measurements. Note that in Fig. 2a, the GFP spectrum has its intensity divided by 100 times, which means that it has a much stronger emission than all other FPs. The anti-Stokes fluorescence excitation process is a universal phenomenon that is not limited by the combination of excitation laser and FPs mentioned above. As long as the absorption peak of the FPs is within a certain range to the excitation laser, there is a huge flexibility for the choice of excitation wavelengths and FPs (Sup. 1). These facts already suggest that the fluorescing mechanism we report here is not a multiphoton excitation scheme of the ultraviolet absorption peak of FPs as one of our previous reports indicated^29^.

To further identify the fluorescing mechanism of the FPs, we verified their fluorescence intensity response according to the excitation intensity. In Fig. 2b and c, the logarithm plot clearly showed a slope at around 1 for both ECFP and mTFP (GFP is not tested because its fluorescence signal easily saturates). This result is a strong evidence that both ECFP and mTFP are excited in a single photon excitation manner, even though their emission peaks are at the wavelength region shorter than the excitation laser light. It is especially worth mentioning that a cw laser can be used for the anti-Stokes fluorescence excitation of FPs, thus the same principle can be readily adaptable to any existing fluorescence microscopes to increase the number of imaging channels^26^.

### Hybrid fluorescence-Raman imaging of cells

**Figure 3:**
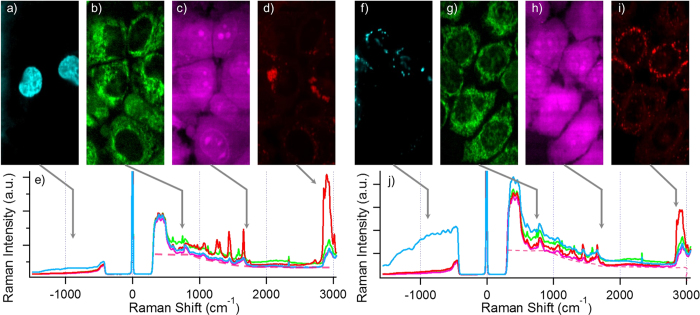
(**a**∼**e**) are the anti-Stokes fluorescence image (**a**), resonance enhanced 750 cm^−1^ cytochrome *c* Raman image (**b**), 1680 cm^−1^ amide I Raman image that shows protein contrast (**c**), 2852 cm^−1^ CH_2_ stretch Raman image that shows long chain lipids (**d**), and the hybrid spectrum taken from a random pixel of the corresponding contrasts (**e**). Cyan is the spectrum with strong CFP emission, green is with strong cytochrome c contrast, magenta from strong protein contrast and red from strong lipid contrast. Note that all these information are acquired within a single scan of the sample. (**f**∼**j**) are the respective images and spectrum of B4GalT1-mTFP labelled HeLa cells as described earlier. All 3 repeats of successfully transfected histone-ECFP HeLa cells and all 6 repeats of successfully transfected B4GalT1-mTFP HeLa cells showed successful combination of fluorescence and Raman imaging as shown here. Also, please note that the presented spectra are without fluorescence background subtraction, but the Raman images are with background subtraction calculations. The width of all images are 40 µm.

**Figure 4:**
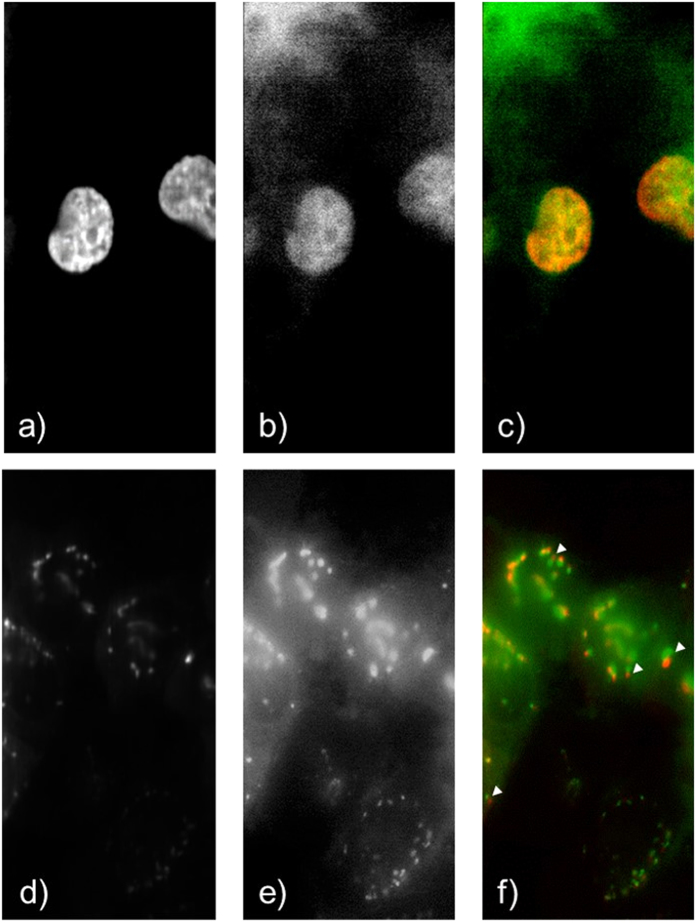
(**a**,**b**) are the respective anti-Stokes fluorescence image and wide field normal fluorescence image of histone-ECFP labelled HeLa cells, and (**c**) is the colour merged image of (**a**), red channel, and (**b**), green channel. (**d**–**f**) are the respective anti-Stokes fluorescence image, wide field normal Stokes fluorescence image and colour merged image of B4GalT1-mTFP labelled HeLa cells. (**d**) is the red channel and (**e**) is the green channel in (**f**). (**a**,**d**) shows much lower background signal than (**b**,**e**) because the anti-Stokes images are taken in a slit-scan manner, which uses a slit to reject out-of-focus background signals, while (**b**,**e**) are wide field images without any restrictions in the detection path. It is worth noting that some of the Golgi bodies are displaced between (**d**,**e**), as clearly shown by the white arrows in (**f**). (**f**) is merged by optimising the contrast overlap in the top left and bottom right cell. The width of all images are 40 µm.

After demonstrating the feasibility of anti-Stokes fluorescence excitation in purified FP systems, next is to apply the method for the hybrid fluorescence-Raman imaging of HeLa cells that express different FP fusion constructs. Cross-talk free anti-Stokes fluorescence image and cytochrome *c*, protein and lipid Raman image HeLa cells were successfully obtained from HeLa cells transfected with histone-ECFP (Fig. 3a∼e) and B4GalT1-mTFP (Fig. 3f∼j). Histone is the protein that help pack the DNA inside cell nucleus^30^. B4GalT1 encodes the β-1,4-galactosyltransferase 1 that helps the synthesis of poly-N-acetyllactosamine, and localise in the trans-Golgi network^31^. The fluorescence contrast in Fig. 3a and f corresponded well to those of histone and B4GalT1, i.e. cell nucleus and Golgi body respectively. The presented spectra have been processed by SVD denoising, and the anti-Stokes fluorescence images were composed by using the denoised hyperspectral dataset. The different colour spectra presented in Fig. 3e and j were selected from a representative pixel within the corresponding colour images (Fig. 3a∼d and f∼i) of the hyperspectral datasets. To verify whether the anti-Stokes excitation of FPs bring unexpected effects to the fluorescence image, we have also compared the two anti-Stokes fluorescence image with the standard wide-field Stokes fluorescence image of the specimen. The result proved that although the fluorescing mechanism is different, both FP imaging methods give almost identical imaging contrast (Fig. 4). However, slight differences in FP localisation can be observed between the two B4GalT1-mTFP images (Fig. 4f). This is because the standard fluorescence image was taken before the hybrid fluorescence-Raman image, and the movement of Golgi bodies within the short time was recorded. This result also highlights the importance of hybrid imaging. Since living cells are dynamic samples that constantly change their morphology and chemical state, if any time lag exists between the fluorescence and Raman measurements, the accuracy of further chemical analyses according to FP expression pattern would be significantly lowered. Our hybrid imaging technique ensures that the fluorescence and Raman data are fully synchronised, and eliminate such uncertainties.

Another concern regarding the anti-Stokes excitation of FPs in live-cell imaging is how the Stokes emission tail of the anti-Stokes fluorescence emission (Fig. 2) would influence the Raman spectra in the hybrid fluorescence-Raman hyperspectral dataset. To address this issue, first, we compare the spectra taken at the subcellular region with and without anti-Stokes fluorescence signals (Fig. 3e and j, spectra in cyan vs. the spectra with other colours). In histone-ECFP transfected cells (Fig. 3e), the spectrum in cyan showed a very weak anti-Stokes fluorescence peak that is barely stronger than the other spectra at the anti-Stokes side. At the Stokes side, the overall background for the spectrum in cyan is almost identical to the cytosol spectrum (in magenta) across 500~3000 cm^−1^, indicating that Stokes ECFP emission tail has negligible effect to the Raman spectra. In the case of B4GalT1-mTFP transfected cells (Fig. 3j), the spectrum in cyan shows a much stronger anti-Stokes fluorescence emission than ECFP in Fig. 3e. Due to the strong anti-Stokes fluorescence intensity, even at the Stokes side, the spectrum in cyan has a significantly higher background than the cytosol spectrum (in magenta), which is around the level of the mitochondria spectrum (in green). This indicates that for ECFP and mTFP, the influence of their Stokes emission tail to the cell Raman spectra is at most around the level of autofluorescence background fluctuation in the cells, which can be easily eliminated by background subtraction algorithms. To more convincingly demonstrate the small effect of the Stokes emission tail of the FPs, we compared the hyperspectral images constructed by the anti-Stokes fluorescence emission band, cytochrome *c* Raman band and fluorescence background at the silent region of the Raman spectra (Sup. 2). The completely distinct imaging contrast between the anti-Stokes fluorescence contrast and Stokes fluorescence background contrast clearly demonstrate that the main contributor for the two fluorescence signals come from different origins. The high similarity of the Stokes fluorescence background contrast to mitochondria, imaged by the cytochrome *c* contrast, further suggests that the main contributor of the Stokes fluorescence background is indeed autofluorescence^32^.

To minimise the background influence on the Raman images, the Stokes region of the hyperspectral dataset were extracted and an automated autofluorescence background subtraction method^33^ was applied before the Raman images were reconstructed (Fig. 3b∼d and g∼i). The cytochrome *c*, protein and lipid Raman images in Fig. 3 all correspond well to our previously reported Raman images of the corresponding vibrational modes^34^,^35^, with minimal cross-talk from the anti-Stokes fluorescence image of the same hyperspectral dataset. This indicates that our hybrid fluorescence-Raman imaging technique indeed provides authentic fluorescence and Raman information for further analysis. The proper combination of FPs and excitation laser is the key of this technique. For example, even under the 532 nm anti-Stokes excitation scheme, the fluorescence signal from GFP is so strong that it overwhelms the Stokes region of the spectra and severe fluorescence signal contamination can be seen in the Raman images (Sup. 3). However, as demonstrated in Sup. 1, by using a different excitation wavelength, it is still possible to use any FP of interest for hybrid imaging. Beside FPs, we have also verified that our method can be applied to chemical probes such as CellTracker (Sup. 4). The hybrid fluorescence-Raman imaging technique is therefore a universal technique that can be applied to most fluorescence imaging studies of living cells to provide the general chemical state of the cell.

### The chemical analysis of stem cells according to Oct-4 expression

Being able to image fluorescence and Raman contrasts at the same time means that the setup enables us to correlate the chemical profile of the cell to its protein expression pattern for the first time. Since our previous studies have successfully visualised the chemical evolution of stem cells throughout their differentiation process^4^,^36^, here we use stem cell differentiation again as the model system to verify whether our technique is sensitive enough to detect the chemical difference between stem cells across subtle differentiation steps. Transcription factor Oct-4 play a key role in maintaining stem cell pluripotency^37^. Mouse embryonic stem (ES) cells cultured in medium with leukaemia inhibitory factor (LIF) maintains pluripotency and show high expression level of Oct-4. Once LIF is removed from culture medium, ES cells begin to differentiate with reduced Oct-4 expression^37^. Here, we established a reporter gene system that uses Oct-4 promoter to drive TFP expression. For ES cells containing the reporter, TFP fluorescence emission would be observed before differentiation, and disappear after they lose their pluripotency^38^.

**Figure 5:**
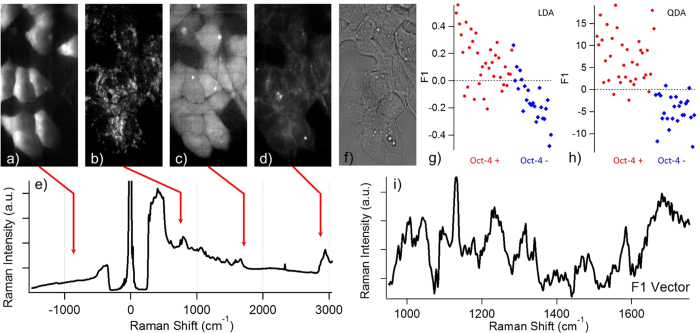
(**a**∼**e**) are the anti-Stokes fluorescence image (**a**), resonance enhanced 750 cm^−1^ cytochrome *c* Raman image (**b**), 1680 cm^−1^ amide I Raman image that shows protein contrast (**c**), 2852 cm^−1^ CH_2_ stretch Raman image that shows long chain lipids (**d**), and the hybrid spectrum taken from a random pixel that strong anti-Stokes fluorescence signal can be seen in the field of view of mouse ES cells transfected with pOct4-mTFP. (**f**) shows the wide field optical image of the ES cells. By comparing (**a**,**f**), it is clear that some cells in the colony express Oct-4 while the others do not. (**g**,**h**) shows the DAPC result for ES cells expressing (Oct-4+) and not expressing (Oct-4−) Oct-4 using linear (**g**) and quadratic (**h**) discrimination algorithms. Each spot represents the averaged spectrum from one cell. DAPC is able to roughly discriminate ES cells with different Oct-4 expression pattern with a 25.71% and 16.67% error rate in (**g**) and a 8.57% and 8.33% error rate in (**h**). (**i**) shows the F1 vector that represents the most dominant spectral difference between the Oct-4+ and Oct-4− groups. The width of all images are 40 μm.

The hybrid Raman-fluorescence imaging of ES cells containing the reporter was conducted after LIF was removed from the culture medium for 3 days to let the cells enter their early stage of differentiation. The hyperspectral hybrid fluorescence-Raman datasets of the ES cells again showed negligible cross-talk between the fluorescence and Raman images (Fig. 5a∼e). However, this time a clear photobleach effect of the mTFP signal can be seen (Fig. 5a). Considering the really low absorption cross-section of mTFP under this off-peak illumination condition, such obvious photobleaching phenomenon was unexpected. The reason behind the unexpected photobleaching rate might be due to the overlap of the incident laser to both the absorption and emission spectra, which resulted in the stimulated cycling between the fluorophore excited state and ground state. It is possible to reduce the laser intensity to minimise such effect (Sup. 5), but the signal-to-noise ratio of the Raman spectra will also decrease, thus reduce the quality of the chemical information presented in the Raman spectra. Comparing the anti-Stokes fluorescence image and transmission optical image of the ES cells (Fig. 5a and f), we show that the ES cells have come to the differentiation stage that even within the same colony, part of the cells has already lost their pluripotency (hence the inactivation of Oct-4) and the other part of the cells are still in the course of differentiating (hence the expression of mTFP through an active Oct-4 promoter).

Since our new hybrid setup can simultaneously acquire the Raman spectra of these ES cells, we continued to verify whether the Stokes Raman spectra is sensitive enough to identify the subtle chemical difference between these ES cells with different expression pattern of Oct-4, but at close differentiation steps. The spectral analysis method we choose for the purpose is the discriminant analysis of principle components (DAPC)^39^,^40^. The linear discrimination plot showed a 25.71% error rate to correctly categorise an Oct-4 expressing (Oct-4+) cell, and a 16.67% error rate to correctly categorise an Oct-4 non-expressing (Oct-4−) cell (Fig. 5g); while the quadratic discrimination plot showed a 8.57% error rate to correctly categorise an Oct-4+ cell, and a 8.33% error rate to correctly categorise an Oct-4− cell (Fig. 5h). To further confirm the statistical significance of the result, we applied Wilks’ lambda test to our result and got a p-value of 0.0087%. This demonstrates that our method can indeed guide Raman spectroscopy to discriminate the tiny chemical shift related to the on-and-off of a specific gene, in this case Oct-4. Furthermore, during DAPC calculation, an F1-vector is drawn between the centres of the two groups in the PC hyper-space. This F1-vector is considered to represent the largest spectral difference in between the two groups after all the meaningful PCs have been taken into account (Fig. 5i). The F1-vector is basically fluorescence background free, suggesting that the fluorescence emission tail at the Stokes region is not the key factor that separates the 2 groups. However, considering that some biomolecules may be more abundant in Oct-4+ cells and others more abundant in Oct-4− cells, the spectral profile of the F1 vector would be a mix of positive and negative Raman spectral components from these differences, making the F1 vector difficult to interpret. More chemical details might be revealed with the further advancements in multivariate spectral analysis techniques to help resolve the detailed spectral components in the F1 vector.

### Chemical analysis of the fluorescence labelled sub-cellular compartments

**Figure 6:**
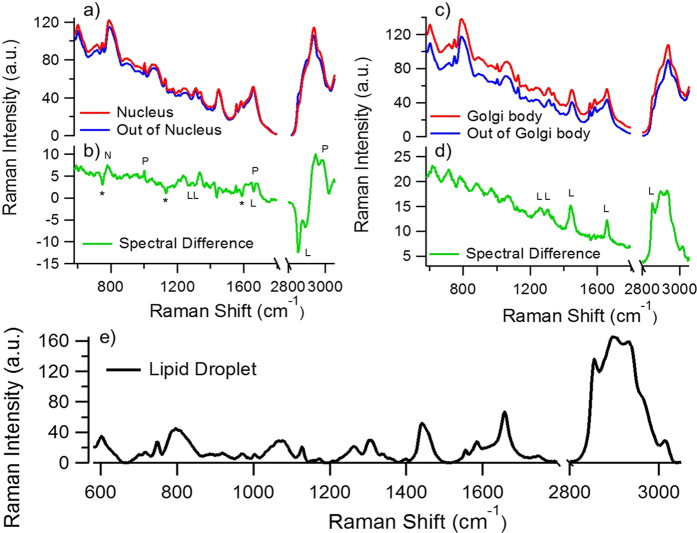
(**a**) Compares the averaged Raman spectra in the histone-ECFP expressing region and the other cellular area in Fig. 3a. The histone-ECFP expressing region is basically equivalent to cell nuclei. Their spectral difference is shown in (**b**). The * mark labels the negative cytochrome *c* peaks, while L, P, N labels the lipid, protein and nucleic acid Raman peaks, respectively. (**c**) compares the averaged Raman spectra in B4GalT1-mTFP expressing region and the other cellular area in Fig. 3f. The B4GalT1-mTFP expressing region is basically equivalent to Golgi body. Their spectral difference is shown in (**d**). The Ls label the lipid Raman peaks that could also be identified in (**b**). (**e**) Shows the Raman spectrum of a lipid droplet in the hyperspectral dataset of Fig. 3f∼j. All spectral features seen in (**d**) can be seen in (**e**), causing the spectral difference difficult to extract by multivariate analysis means.

Besides the chemical profiling of living cells according to protein expression pattern, another important application of the hybrid imaging technique is to use FPs as a guide for the chemical analysis of living cells. Here, we use the hybrid fluorescence-Raman images of histone and B4GalT1 labelled HeLa cells in Fig. 3 as example. First, about the histone-labelled HeLa cell in Fig. 3a, the averaged Raman spectra within and outside of the labelled cell nuclei in Fig. 3a can be easily extracted (Fig. 6a). For the averaged Raman spectra of the non-fluorescing compartment, the pure culture medium spectra that does not contain any cellular information are excluded to ensure we are comparing the spectral content within the cellular region. It is not difficult to see that even though cell nuclei are considered to have a significant difference in its nucleic acid content compared with cytosol, the general morphology of the nucleus spectrum is still somehow similar to the averaged spectrum of the out-of-nucleus region (Fig. 6a). The detailed chemical differences can be more easily identified by calculating the difference spectrum. By subtracting the nucleus spectrum with the out-of-nucleus spectrum (Fig. 6b), we can easily identify that the cytochrome *c* Raman bands (750 cm^−1^, 1132 cm^−1^, 1587 cm^−1^) and lipid Raman bands (1266 cm^−1^, 1302 cm^−1^, the sharp 1656 cm^−1^ peak, 2852 cm^−1^) point to the negative direction, while the protein Raman bands (1004 cm^−1^, the broad 1656 cm^−1^ band, 2950 cm^−1^) and nucleic acid Raman band (780 cm^−1^) points towards the positive direction. The result corresponds to our general understanding that cell nucleus is rich in protein and nucleic acid content, while having less cytochrome *c* and lipid content than the other cellular compartments.

Next, we used the same method to analyse the chemical content of Golgi body in living cells. It is worth mentioning that no unsupervised multivariate spectral analysis is able to discriminate Golgi body from its surroundings yet. The averaged Raman spectrum of the fluorescing compartment in B4GalT1-mTFP expressing cells (Fig. 3f) and the non-fluorescing area were calculated (Fig. 6c). Again, the two spectra look highly similar to each other at the first glance. To visualise the subtle chemical difference, the difference spectrum between the Golgi body and the other compartments is calculated (Fig. 6d). The broad background in the difference spectrum most likely originates from the mTFP fluorescence tail in the Stokes region, and the wave-like structure below 1100 cm^−1^ is most likely due to the spectral profile of the notch filter. Such wave-like spectral pattern does not appear when the spectral difference is below 15 CCD counts (Sup. 6). Above 1100 cm^−1^, the spectral profile clearly shows the spectrum of lipids. The 1266 cm^−1^, 1302 cm^−1^, 1656 cm^−1^, and 2852 cm^−1^ Raman bands can all be seen in the negative spectral component in the nuclei difference spectra as well (Fig. 6b). Together with the 1440 cm^−1^ Raman band, these can all be assigned to lipid bands. Since the spectral difference is similar to the Raman spectrum of lipid droplets (Fig. 6e), but at a much lower contrast, it is very difficult to extract the information by multivariate analysis means. Repeat experiments show consistent lipid-like difference spectrum for Golgi body analysis, while many of them also exhibit the Raman spectral features of cytochrome *c* (Sup. 6). This indicates that Golgi body might also have a tendency to attach to mitochondria in HeLa cells.

## Discussion

Hybrid fluorescence-Raman microscopy has enormous potential to reveal the ongoing chemistry in living cells, especially when combined with FP imaging. FPs have been widely used to indicate protein localisation, expression dynamics, and the physiological state of cells^18^,^41^. Reporter constructs that express FP by the promoter of interest is a common technique to visualise the expression status of various marker genes in cells^16^. The translocation of FP-tagged transcription factors is also a widely used indicator of the activation of regulatory pathways^42^. There are even combinations of FP constructs that are designed to present different colours according to different cellular states^17^. All these techniques serve as powerful tools to study cellular regulation through protein signalling pathways. With our newly developed hybrid fluorescence-Raman imaging system, we are able to further correlate these protein expression pattern to the chemical behaviour of cells during biological events. In fact, we have applied the hybrid imaging technique for the correlation of stem cell chemical profile according to Oct-4 expression pattern. Such real-time chemical analysis according to protein expression pattern in living cells can hardly be done by any other methods. Further extension of this study may lead to the label-free determination of cellular differentiation state, which is important for the quality control of stem cells for regenerative medicine purposes. Ideally, if the chemical response of cells over all signalling checkpoints can be identified, we will be able to determine the current physiological state of a cell simply by taking its Raman spectrum, without the need of any fluorescence labelling treatments. This is especially important in the development of all kinds of cell therapies, because for cells that are going to be transplanted into human patients, it would be preferable to avoid transfection with FPs for the visualisation of cellular states.

In addition to the chemical profiling of cells with different protein expression patterns, another important application of the hybrid imaging technique is the chemical analysis of fluorescence-labelled sub-cellular compartments in living cells. Similar studies have been conducted by unsupervised multivariate data analysis of hyperspectral Raman datasets without any fluorescence aids^43^,^44^. However, the outcome is seldom biologically convincing. The reason is most likely due to the limit of current multivariate data analysis methods. Most of these methods focus on the analysis of the general spectral morphology, but the chemical composition of different sub-cellular compartments mostly differ only in the minor components. For example, except for densely packed organelles such as nucleus or lipid aggregates, which are always very easily discriminated by multivariate Raman spectral analysis, the chemical composition of the other organelles are most likely to have a similar ratio of water content (considered to be more than 70% of the total weight in cells) and total protein content (considered to be more than 50% of dry weight in cells) to each other, which makes them extremely difficult to be distinguished between each other by the multivariate analysis of Raman datasets. For the visualisation of endoplasmic reticulum and Golgi body by Raman related methods, fluorescence-guided multivariate Raman spectral analysis has been reported in fixed cells^45^. However, our hybrid imaging method is the first of its kind that can be applied to living cells, and has the potential to precisely keep track of the subtle chemical differences between the labelled and unlabelled compartments in real-time during biological experiments.

The applicable target of this hybrid imaging method is far beyond what is presented in this report. Basically, the chemical response of all biological checkpoints that could be visualised with FP expression patterns could be extracted with this technique. Not only will this technique benefit the clinical application and field study of cells that cannot be labelled by fluorescent probes, once the complicated Raman spectral profile can be deciphered, it will also contribute to our understanding in the fundamentals between protein signal cascades and the metabolome perturbation in cells. Since the key of this hybrid imaging method is to find the ideal combination between the excitation wavelength and the fluorescent protein, it is also possible to shift the excitation wavelength to near infrared region and use red or infrared fluorescent proteins for the studies of specimen with strong autofluorescence properties, such as *in vivo* experiments on mouse models. Further advances in multivariate spectral analysis techniques will also help boost the extractable chemical information from the complicated spectra. In addition, the anti-Stokes fluorescence principle might also be used to combine fluorescence imaging with infrared spectroscopy or surface-enhanced Raman scattering techniques, which will give different chemical information with a higher signal-to-noise ratio (S/N) than traditional Raman spectroscopy. The combination with the temporal discrimination of fluorescence and Raman scattering^46^,^47^,^48^, which was proposed to reduce the fluorescence background, may also be used to suppress the fluorescence background further in order to expand the choice of fluorescence probes for the proposed methods. We believe that this technique will help connect the missing link, and contribute to the further advancement in the chemical analysis of living cells.

## Methods

### Hybrid fluorescence-Raman microscopy

The configuration of the experimental setup and the imaging protocol is mostly the same as our previously reported slit-scanning Raman microscope^49^. The same setup has been used for the time-lapse study of osteoblastic mineralisation process over 3 days’ time, suggesting the 532 nm laser wavelength and the measurement condition we choose here biologically safe^50^. The major difference is that we changed the edge filter in the setup to a notch filter, and the grating angle in the spectrometer was set at an angle that the incident wavelength is close to the centre of the CCD so that both the anti-Stokes and Stokes side of the spectrum can be collected simultaneously. It is also important to have 2 notch filters before the spectrometer to block the strong Rayleigh scattering from the sample. We have also confirmed that the hybrid fluorescence-Raman detection can be applied to another home-built confocal Raman microscope by using notch filters instead of edge filters in the detection path.

As for the measurement condition for each figures, the histone-ECFP and B4GalT1-mTFP HeLa cell hyperspectral dataset in Figs 3 and 4 are taken at 1.5 mW/μm^2^ laser intensity, 1.5 seconds accumulation time for each line and 500 μm scan step over the horizontal axis of the images. The typical acquisition time for the presented hyperspectral datasets was around 2 minutes. The pOct-4-TFP ES cell hyperspectral dataset in Fig. 5 was taken at 3 mW/μm^2^, 5 seconds accumulation time for each line and 500 μm scan step over the horizontal axis of the images. Since the acquisition time is longer than in Figs 3 and 4, the acquisition time for the dataset was around 7 minutes.

### Fluorescent protein emission spectra and response curve measurements

The purified BFP, ECFP, mTFP and GFP solutions were kindly provided by Prof. Takeharu Nagai at Osaka University and published in a previous report^29^. The anti-Stokes emission spectra were obtained by the hybrid fluorescence-Raman microscope as described above. Since the setup we used has a slit-scanning configuration, the spectra we present in this paper are the averaged spectra over the whole slit range. The excitation wavelength was 532 nm, laser intensity at 2.3 mW/μm^2^ and the acquisition time was 5 seconds for the BFP, ECFP and mTFP spectra. As for the GFP spectrum, the laser intensity was 0.15 mW/μm^2^ and the acquisition time was 0.25 second. The illumination condition was about 300 times milder for GFP than the other measurements. To keep the GFP spectrum stronger in intensity when plotted together with the other FPs, the intensity of the GFP spectrum was then multiplied by 3 when shown in Fig. 2a.

As for the response curve, the laser intensity was manually controlled by a half-wave plate and a Glan-laser prism. The intensity values used in both the ECFP and mTFP response curves are the fluorescence intensity at the 498 nm pixel.

### Cell culture and preparation

Both the HeLa and ES cell lines are the same ones used in our previous researches and cultured in the same condition^4^,^34^. The cell lines have not been authenticated and tested for mycoplasma contamination during the period of this study. For the HeLa cell hybrid imaging, histone-ECFP and B4GalT1-mTFP plasmids were also provided by Prof. Takeharu Nagai at Osaka University and published in a previous report^29^. Transfection was conducted by electroporation of the HeLa cells 2 days before the measurement. For the detailed procedure, a confluent 10 cm dish (Corning) of HeLa cells were trypsinised by 0.25% trypsin-EDTA (Gibco) and span down by a Kubota 2100 centrifuge (the condition for all centrifugation in this paper is 800 rpm, 5 min). The cells were then washed once with HEPES buffer (Gibco), spun down again, then resuspended by 500 μl HEPES buffer. 100 μl of the cell suspension was then transferred to a cuvette for electroporation, mixed with 5 μl of 1 mg/ml plasmid of interest, and electroshocked by an electroporator (NEPA21 Type 2, NEPA GENE). The poring condition is 2 pulses at 115 V, 7.5 ms pulse length, 50 ms pulse interval and 10% decay rate; and the condition for transfer is 20 V, 50 ms pulse length, 50 ms pulse interval and 40% decay rate. Afterwards, the 100 μl suspension was immediately transferred to two 3.5 cm culture dishes (Corning) each containing a clean quartz coverslip and 2 ml of DMEM (SIGMA) supplemented with 10% FBS (Hyclone) and 1% Strep Pen (Gibco). After 2 days of culturing under 37.5 ˚C, 5% CO2, the quartz coverslips with attached HeLa cells were used for hybrid fluorescence-Raman measurements.

To make an Oct4-TFP reporter, cDNAs of Oct4 promoter sequence and TFP were amplified with Oct4 reporter used in previous study^51^ and Golgi-TFP construct, respectively, as a template. The fragments were then cloned into PiggyBac Transposon Vector (System Biosciences) with puromycin resistance marker. Mouse ES cells were maintained in DMEM medium (Sigma-Aldrich) supplemented with 10% FBS, 1% penicillin (Sigma-Aldrich), 1% streptomycin (Sigma-Aldrich), 1% GlutaMAX-1 (Gibco), 1% non-essential amino acid (Gibco), 1% nucleosides (Millipore), 1% sodium pyruvate (Sigma-Aldrich), 0.1% 2-mercaptoethanol (Sigma-Aldrich), and 0.1% leukemia inhibitory factor (Nacalai). The cells were cultured on 0.1% gelatin-coated polystyrene cell-culture dishes without feeder cells. Transfection of the Oct4-TFP reporter was carried out by FuGENE6 HD (Promega). Antibiotic selection by 0.5 μg/ml puromycin started 2 days after transfection. After 7 days selection, ES cells with TFP fluorescence were isolated by flow cytometry (BD Biosciences).

### Spectral analysis methods

For the spectral analysis of ES cells, we employed DAPC, which utilizes the scores of principal component analysis (PCA) as explanatory variables of discriminant analysis (DA). The procedure was written and performed by IGOR Pro (WaveMetrics). First, cells in the anti-Stokes fluorescence images were classified into two groups, Oct-4-positive (Oct-4+) and -negative (Oct-4−) cells. We used hybrid fluorescence-Raman images of totally 59 cells, within them, 35 cells were classified to Oct-4+ and 24 cells to Oct-4−, respectively. Mean spectra were calculated by averaging the spectra of all pixels in each cell region. The Raman spectra were standardized by subtracting the mean value of each spectrum and dividing the spectrum by the standard deviation of the spectrum. PCA was performed to the set of the mean spectra of all cells including Oct-4+ and Oct-4− cells, which were decomposed as a linear combination of principal component (PC) loading vectors. The top 9 PC vectors were selected for the following calculations (Sup. 7). The non-linear iterative partial least squares (NIPALS) algorithm was utilized for the extraction of PC loading vectors and PC scores. The DA was performed for the PC scores to build a discrimination model. We calculated discriminant scores in the manners of linear DA (LDA) and quadratic DA (QDA) for comparison. The LDA scores are given by the Fisher’s linear discriminant function, corresponding to the projection of the PC scores to the F1 axis, whereas the QDA scores are calculated as the logarithm of ratio of the probabilities that the sample belongs to each of two classes.

As for the cell nucleus and Golgi body spectral analysis, we first applied a binary filter to the anti-Stokes images of each hyperspectral dataset to select the fluorescing area, and categorized these region as our “region of interest” (ROI). Then, we applied the binary filter to the CH stretch images constructed by integrating the signal intensity between 2800~3000 cm^−1^ to select the cell region. The CH stretch image is dominated by protein contrast, so the image contrast is similar to the 1680 cm^−1^ amide I Raman image in Fig. 3c and h that well indicate the whole cell region. We then averaged the Stokes Raman spectra of the image pixels within the ROI to obtain the nucleus and Golgi body spectra. For the out of nucleus and out of Golgi body spectra, we excluded the ROI from whole cell region, and defined the region as “out of ROI”, then averaged Stokes Raman spectra from the “out of ROI” region. The spectral difference is simply calculated by subtracting the ROI spectra with the “out of ROI” spectra. No background-correcting algorithms or denoising methods are applied for the spectral analysis here.

## Additional Information

**How to cite this article**: Chiu, L.-d. *et al*. Protein expression guided chemical profiling of living cells by the simultaneous observation of Raman scattering and anti-Stokes fluorescence emission. *Sci. Rep.* **7**, 43569; doi: 10.1038/srep43569 (2017).

**Publisher's note:** Springer Nature remains neutral with regard to jurisdictional claims in published maps and institutional affiliations.

## Supplementary Material

Supplementary Information

## Figures and Tables

**Figure 1:**
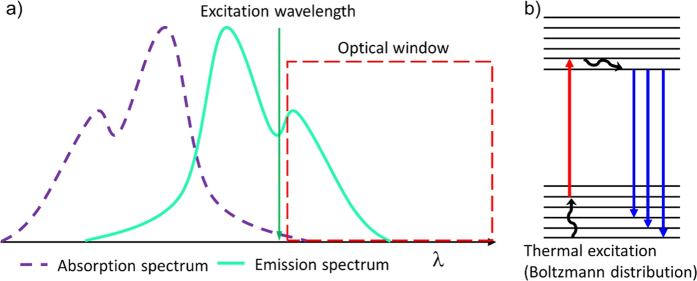
The principle of anti-Stokes fluorescence excitation. (**a**) The fluorophore is excited at its long-wavelength absorption tail and the fluorescence emission peaks comes at the shorter wavelength of the excitation light. It is a single photon process. This leaves an optical window at the longer wavelength region than the incident laser for the combination of other optical techniques, such as Raman spectroscopy. (**b**) The energy diagram for anti-Stokes fluorescence emission. Boltzmann distribution explains the low probability of fluorophores excited to a higher vibrational state in the ground state, thus resulting in anti-Stokes fluorescence by a long-wavelength excitation laser.
