# Supplementary material for: Protein expression guided chemical profiling of living cells by the simultaneous observation of Raman scattering and anti-Stokes fluorescence emission
Source: Sci Rep. 2017 Mar 8;7:43569. doi: 10.1038/srep43569 (PMC5341087; doi:10.1038/srep43569)
Supplement: Supplementary Information [file srep43569-s1.pdf]

Protein expression guided chemical profiling of living cells by the simultaneous observation of Raman scattering and anti-Stokes fluorescence emission

Liang-da Chiu[1,2,*], Taro Ichimura[3], Takumasa Sekiya[2], Hiroaki Machiyama[4], Tomonobu Watanabe[3], Hideaki Fujita[3,4], Takeaki Ozawa[1], Katsumasa Fujita[2,*]

[1]Department of Chemistry, the University of Tokyo, Tokyo, Japan
[2]Department of Applied Physics, Osaka University, Osaka, Japan
[3]Quantitative Biology Center, RIKEN, Osaka, Japan
[4]Immunology Frontier Research Center, Osaka University, Osaka, Japan
Correspondence should be addressed to L.-d. C. (liang-da@chem.s.u-tokyo.ac.jp) or K. F. (fujita@ap.eng.osaka-u.ac.jp)

Supplementary data list

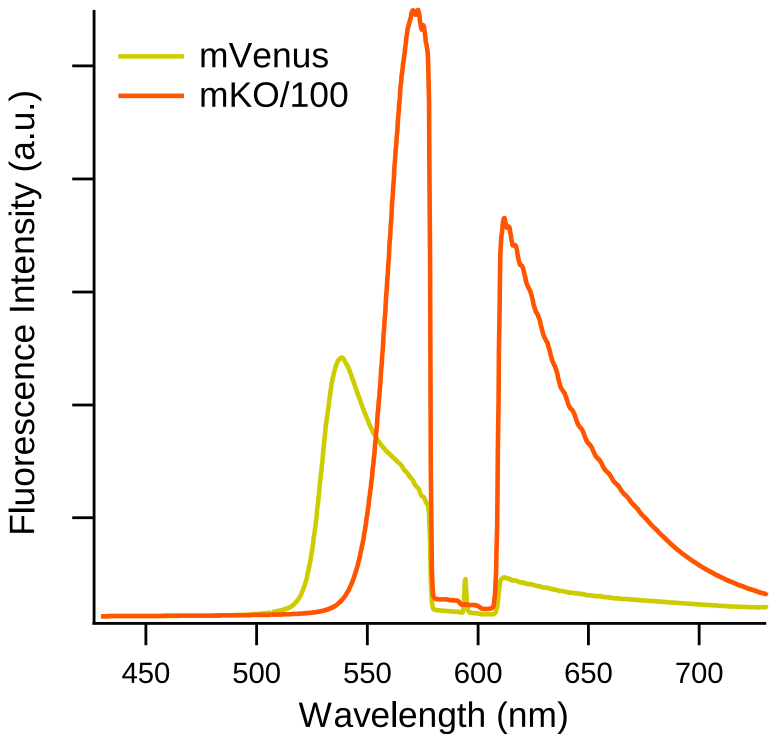

Sup. 1 The anti-Stokes fluorescence emission spectra of mVenus and mKO (Kusabira-Orange) excited by 594 nm excitation laser. The spectral valley around 594 nm is due to the notch filter. Note that the excitation intensity for mKO is 100 times lower than mVenus, because the emission spectrum for GFP would saturate otherwise.

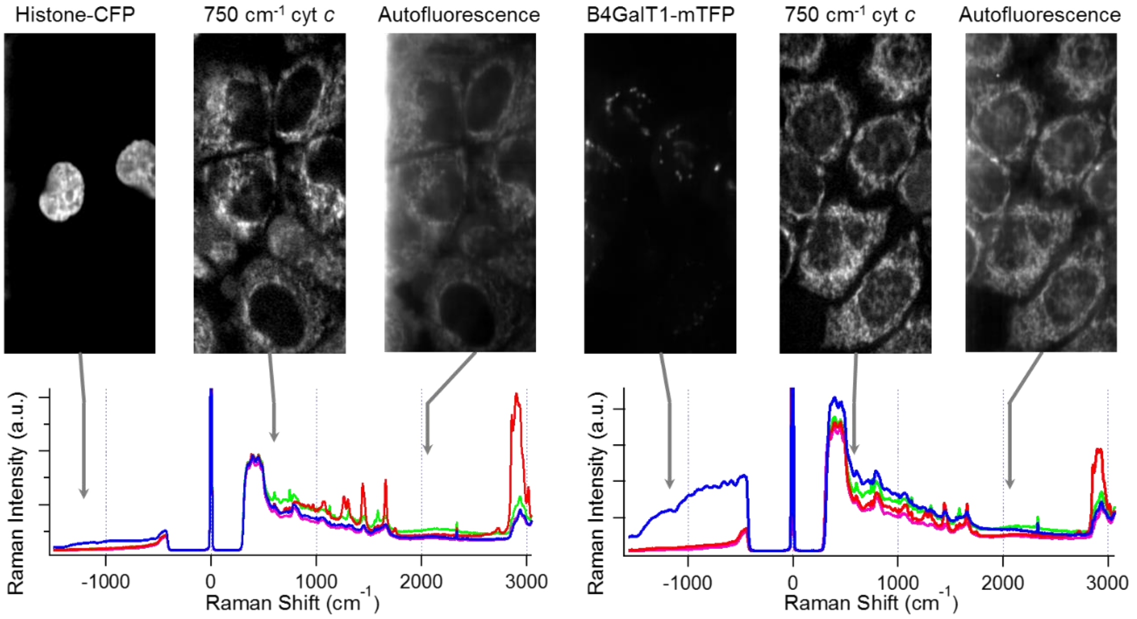

Sup. 2 Two sets of hyperspectral datasets that show the fluorescence background at the Stokes side and the Anto-Stokes side has different origins. The datasets are the same one as used in Fig. 3. Notice that the autofluorescence background at the Raman spectral silent region of both datasets show totally different contrast than the anti-Stokes fluorescence signals, and resembles the mitochondria contrasts as shown by the cytochrome *c* Raman images. This explains that the main contributor of the broad fluorescence background under the Raman spectra is indeed from autofluorescence, instead of the Stokes tail of the anti-Stokes fluorescence emission.

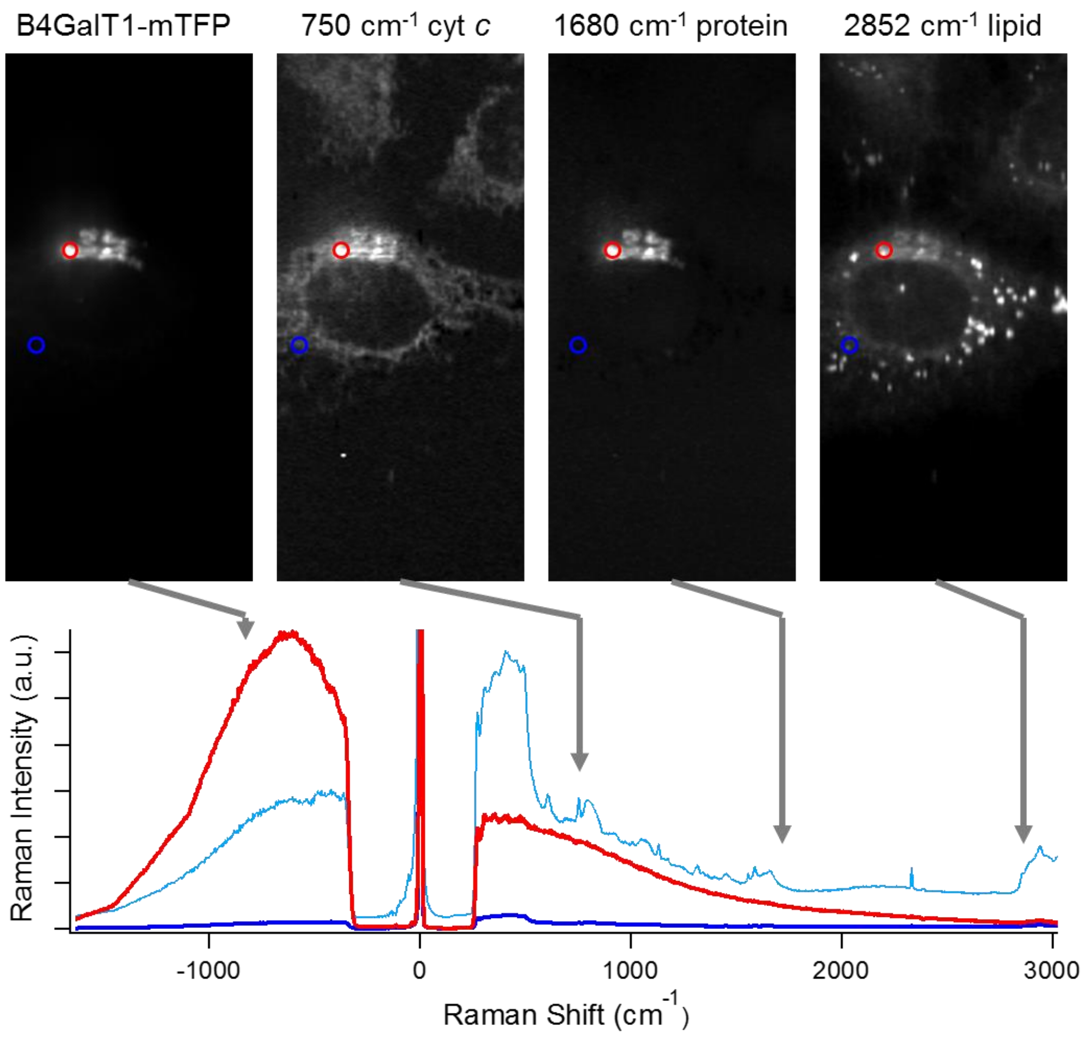

Sup. 3 A hyperspectral Raman dataset of HeLa cell transfected with B4GalT1-GFP. The red spectrum is taken from the brightest fluorescence spot in the anti-Stokes fluorescence image, indicated by a red circle. The spectrum showed almost only fluorescence signal, even in the Stokes region. The dark blue spectrum is the spectrum from the blue circle in the images, which indicates the spectrum of a subcellular compartment that shows no contamination of fluorescence signals in the Raman images. Note the significant intensity between the red and the dark blue spectra. The light blue spectra is the 10 times amplification of the dark blue spectrum to show the detailed spectral features. Serious contamination of fluorescence signals can be seen in all Raman images, making GFP an unsuitable probe for the combination with 532 nm excited Raman spectroscopy.

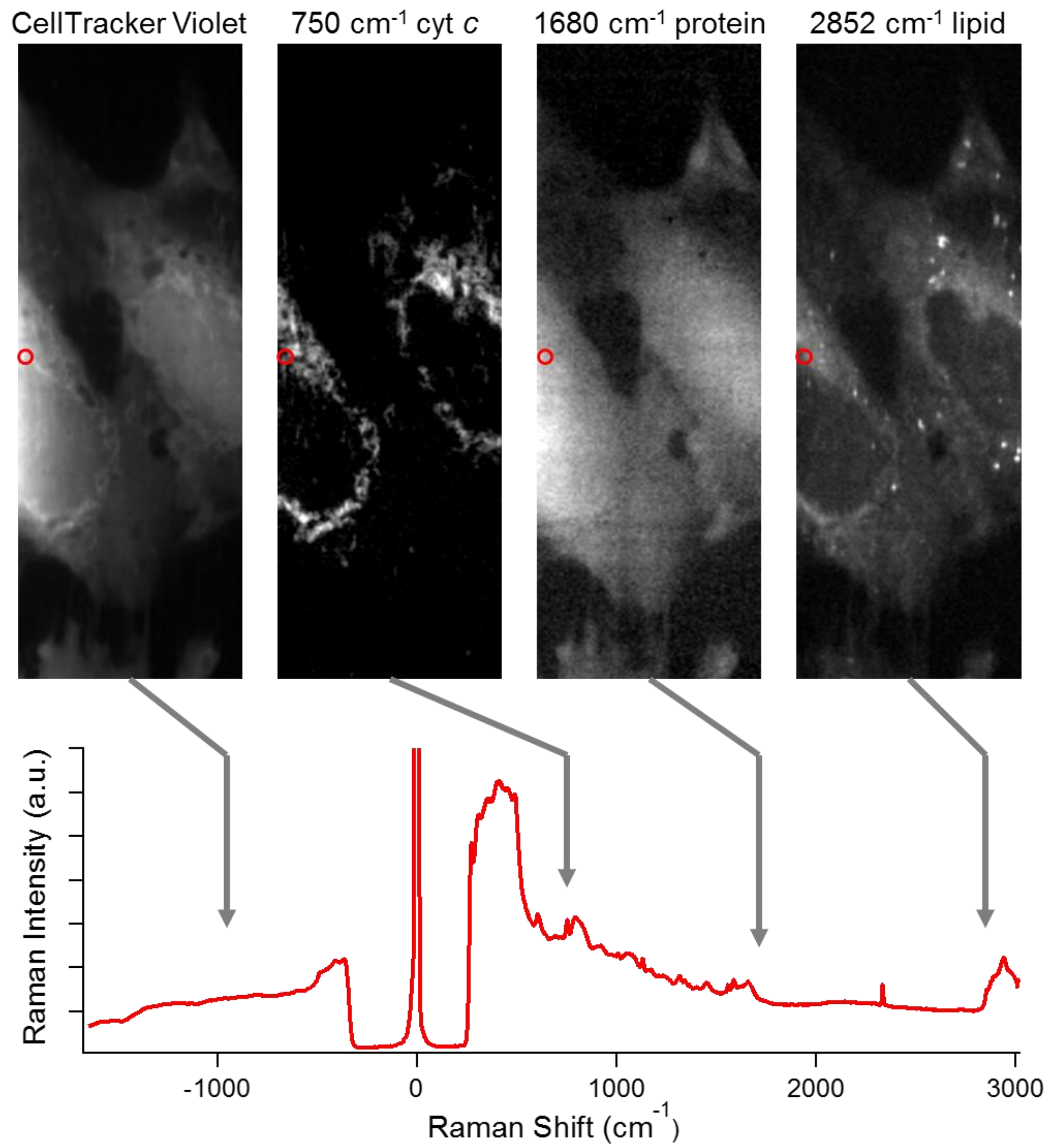

Sup. 4 A hyperspectral Raman dataset of HeLa cell stained with CellTracker Violet BMQC. The spectrum is taken from the brightest fluorescence spot in the anti-Stokes fluorescence image, indicated by a red circle. Crosstalk between the anti-Stokes fluorescence image and the Raman images are not visualised, suggesting organic dyes can also be combined with Raman spectroscopy in the same way.

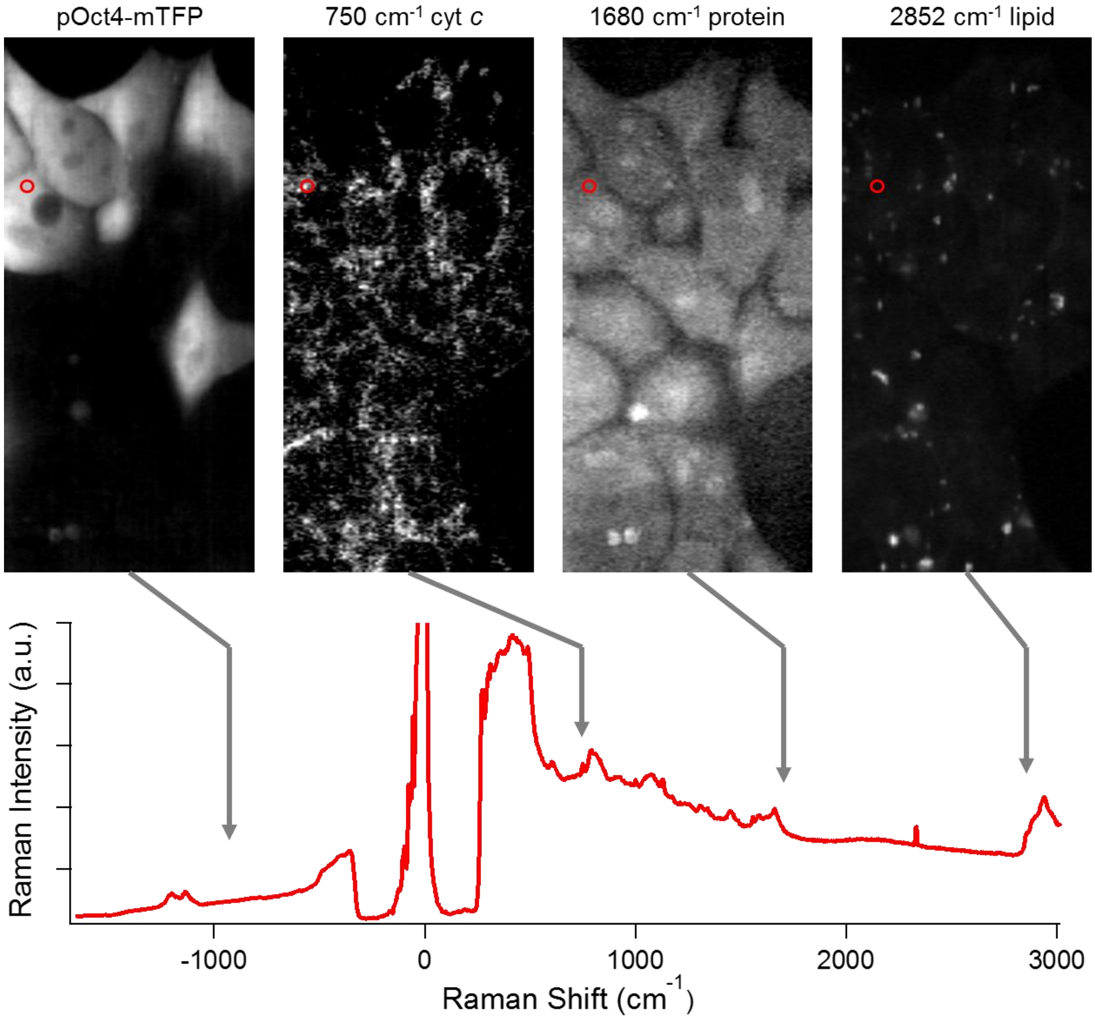

Sup. 5 The same experiment as Fig. 5a~e, only that the excitation condition is different. In this case, the laser intensity is 1 mW/μm², and acquisition time is 5 seconds. Note that the photobleaching effect is largely reduced by decreasing the laser intensity from 3 mW/μm² (Fig. 5a~e) to 1 mW/μm².

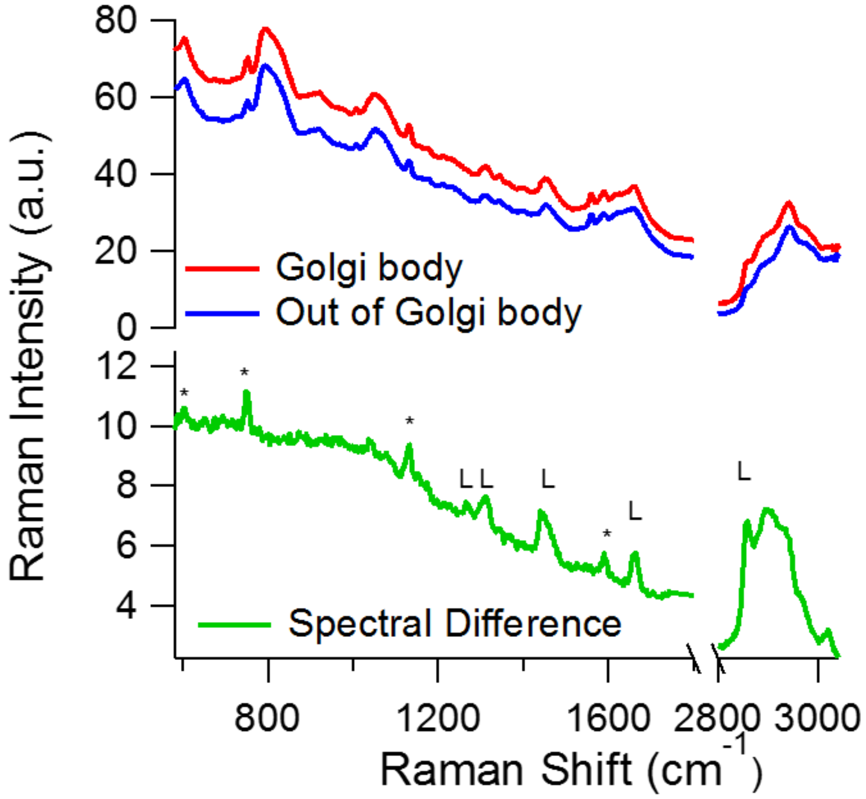

Sup. 6 One of the other Golgi body spectral analysis datasets. Notice that besides the lipid bands that also appears in Fig. 6, as labelled by L in the difference spectrum, cytochrome c Raman bands that are labelled by * are also seen in this dataset. Within the 12 datasets we carefully analysed, Fig. 6d and the difference spectrum here are the only two spectral difference patterns we could observe.

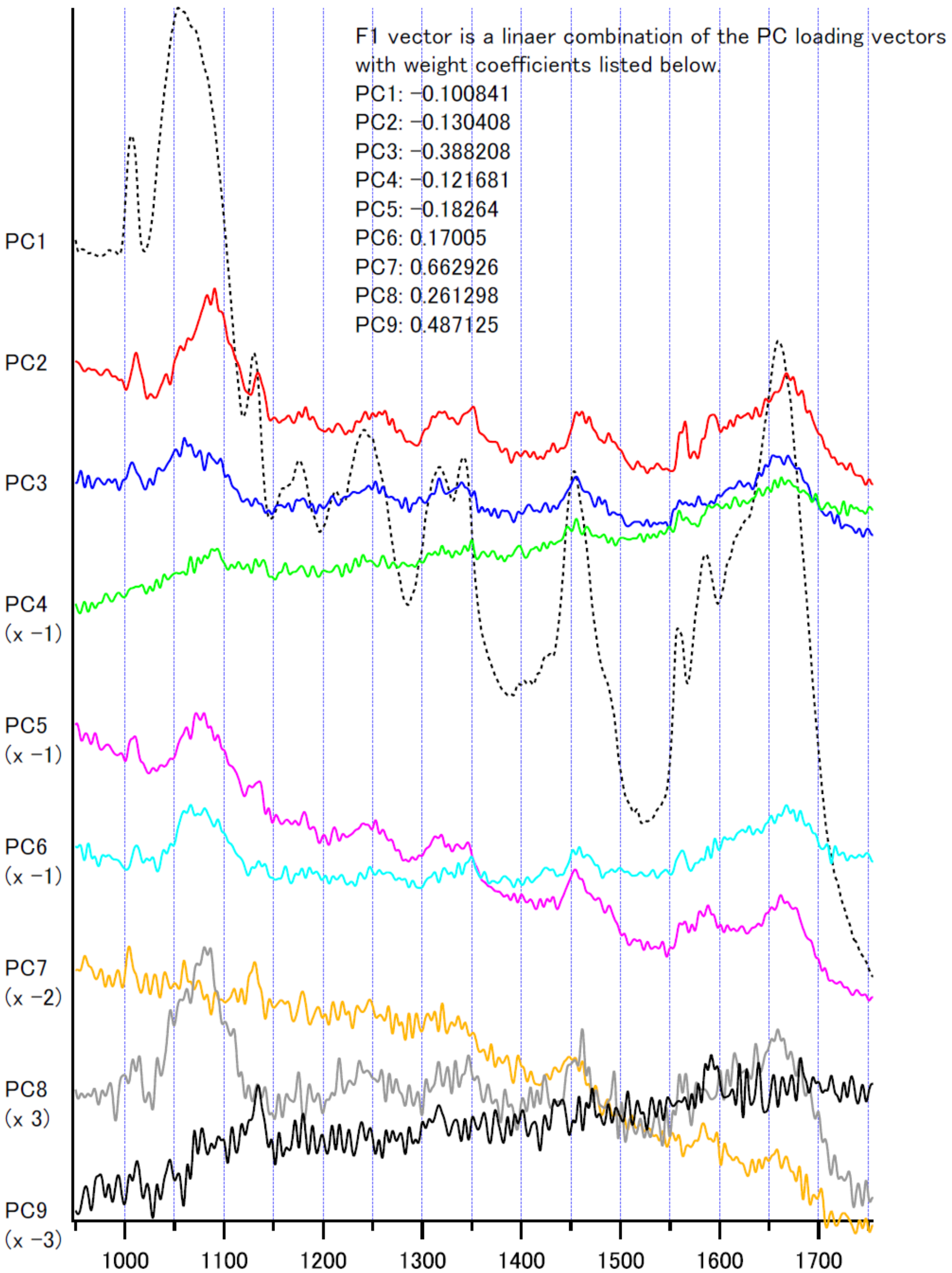

Sup. 7 The top 9 PC vectors used for the DAPC calculation in Fig. 5. The weight coefficients of the PCs that are used to reconstruct the F1 vector is also presented in the figure.
